# Delineating the Antiapoptotic Property of Apigenin as an Antitumor Agent: A Computational and In Vitro Study on HeLa Cells

**DOI:** 10.1021/acsomega.4c01300

**Published:** 2024-05-30

**Authors:** Deepika Bhagavatula, Tarique Noorul Hasan, Huzefa Vohra, Sherareh Khorami, Arif Hussain

**Affiliations:** † School of Life Sciences,Manipal Academy of Higher Education, Dubai 345050 ,United Arab Emirates; ‡ Department of Molecular Genetics, Sh. Tahnoon Bin Mohammed Medical City (STMC), Al Ain, Pure Health, Abu Dhabi 17822, United Arab Emirates

## Abstract

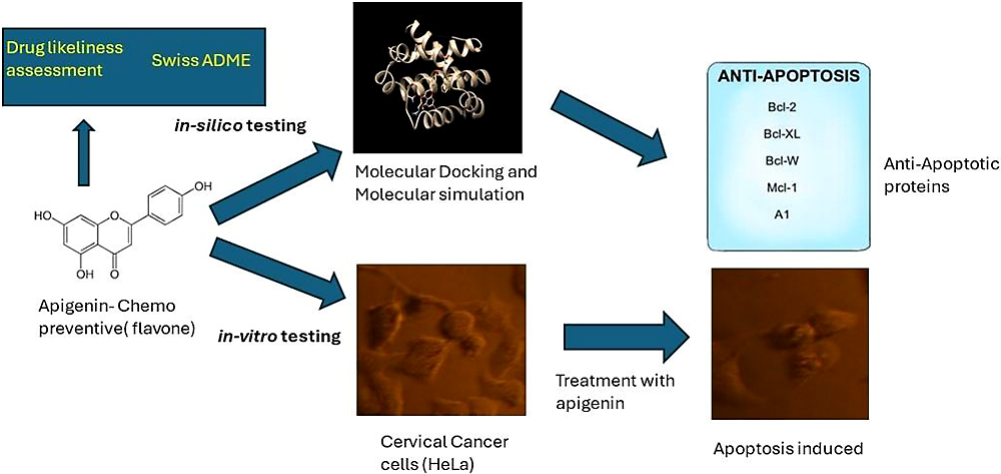

Apigenin, a flavonoid, is reported to have multiple health benefits including cancer prevention; this study evaluates the drug likeliness and Swiss ADME properties of apigenin. Apoptosis, which is a key hallmark of cancer, is associated with the deregulation of the balance between proapoptotic proteins and antiapoptotic proteins such as BCL-2,BCL-xl, BFL-1, BCL-w, BRAG-16, and MCL-1. The docking studies of apigenin with the mentioned proteins was performed to identify the interactions between the ligand and proteins, which suggested that apigenin was able to bind to most of the proteins similar to the inhibitory molecules of its native structure. A remarkable reduction in the total energy after energy minimization of apigenin-antiapoptotic protein complexes suggested increased stability of the docked complexes. The same complexes were found to be stable over a 10 ns period of molecular simulation at 300 K. These findings advocated the study to evaluate apigenin’s potential to inhibit the HeLa cells at 5, 10, and 15 μM concentrations in the clonogenic assay. Apigenin inhibited the colony-forming ability of HeLa cells in a dose-dependent manner over a fortnight. Light microscopy of the treated cells displayed the morphological evidence characteristic of apoptosis in HeLa cells such as blebbing, spike formation, cytoplasmic oozing, and nuclear fragmentation. Thus, these results clearly indicate that apigenin may be used as a potential chemopreventive agent in cervical cancer management.

## Introduction

World Health Organization (WHO) has estimated cancer to be the second deadliest disease in the survey of 183 countries.^1^ Worldwide, the incidence and mortality of cancer recorded is rapidly growing.^1−3^ Cancer defined as “the disease which never heals” has specific characteristics such as nonstop proliferation of abnormal cells as well as evading the immune signals.^4−6^ Traditional treatments such as surgery, radiation, and chemotherapy as well as the latest immunotherapy have all been used in cancer treatment with limited success on complete cure.^4^ The various obstacles created in cancer treatment owing to their key characteristics such as uncontrolled cell proliferation, oppressing cell cycle checkpoints, and evading apoptotic signals along with many more have all been recorded as “Hallmarks of Cancer”.^5^

Tumorous cells do not possess new mechanisms, instead exploit the bodily cellular and molecular pathways and bypass various protective immune responses that prevent their formation and growth.^5−8^ Apoptosis, a key hallmark of cancer which is also known as “Programmed Cell Death”, is a mechanism crucial for normal tissue homeostasis,^3^ and deregulation in this mechanism leads to various serious disease conditions, specifically cancer. Apoptosis is targeted both intrinsically as well as extrinsically via a cascade of triggers. These mechanisms include various proteins which are proapoptotic (i.e., lead the abnormal cell into apoptosis/death) such as BAX, BAK, and BOK (BCL-2 related ovarian killer), BID, BIM, PUMA, NOXA, BIK, BAD, HRK, and BMF as well as antiapoptotic proteins (i.e., lead the cell away from apoptosis) such as BCL-2, MCL-1, A1/Bfl-1, Bcl-B/Bcl2L10, and BCL-xL (BCL extralarge).^3^ BIK, BAD, BID, BIM, BAX, BAK, BOK, BCL-2, MCL-1, A1/Bfl-1, Bcl-B/Bcl2L10, BCL-xL, and BCL apoptotic proteins have been known to be altered transcriptionally, translationally, and post- translationally in various cancers; therefore, inappropriate apoptotic signaling interferes with the genome integrity, and this is one of the crucial hallmarks that contributes to carcinogenesis.^3^

Due to the limitations of conventional methods in complete cancer treatment, research has now begun to explore various naturally occurring compounds or products that may be potential anticancer drugs.^6−9^ Many such natural compounds or their derivatives have been clinically used since 1940s.^9^ These naturally occurring products are also known as flavonoids which are secondary plant metabolites possessing various disease protective abilities such as antiviral, antibacterial, antioxidant, as well as anticancer effects.^6,7,9,10^ There are about 6000 compounds that are found with different chemical structures which are further divided into various groups such as flavanols, flavonols, anthocyanidins, isoflavones etc.^9,11−14^

Apigenin, a flavonoid with a characteristic yellow needle structure having a molecular weight of 270.24 g/mol and abundantly found in vegetables and fruits such as onions, oranges, thyme, etc., has been known to display strong action against cancer.^9,11−14^ Apigenin has been reported to be an effective antitumor agent owing to its minimal toxicity to normal cells and nonmutagenicity compared to its counterparts.^9,15−20^ Furthermore, apigenin has been proven to contribute to the inhibition of other cancer hallmarks such as cell invasion and metastasis by regulating various signaling pathways involved in causing various cancers such as leukemia, breast, pancreatic, lung, ovarian, prostate, etc.^9^ This study is therefore aimed at exploring the anticancer abilities of apigenin against cervical cancer (HeLa) in both in vitro and in silico conditions, proving its effectiveness on cell viability as well as its potential in suppressing antiapoptotic proteins such as BCL-2, BCL-w, BCL-xl, MCL-1, BRAG-1, and BFL-1 which are reported to be upregulated in cervical cancer.

## Materials and Methods

### Preparation of Ligand

The 3D structure of apigenin has been downloaded from PubChem in SDF-3D format. Further, the SDF format was converted to .pdb format by using the SMILES formula in the Open Babel.^21^ For downstream study, it was downloaded and saved as ligand (apigenin.pdb). The tool Chimera 1.16^22^ was used to visualize the 3D structure of the ligand.

### Drug Likeliness Study of the Ligand

**Figure 1:**
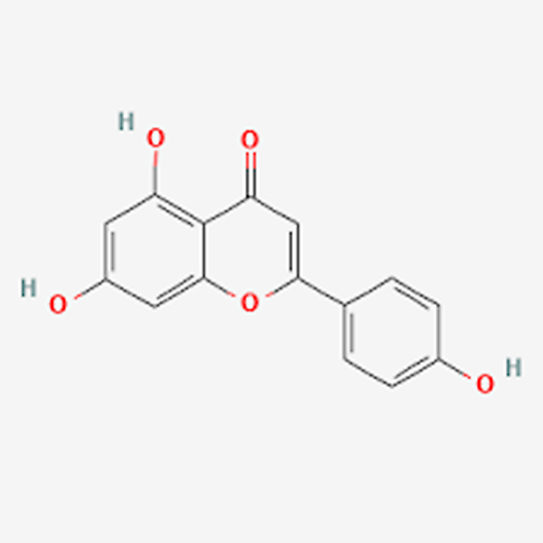
Chemical structure of apigenin (structure adapted from PubChem).

The ligand (apigenin) (Figure 1) was screened for its drug likeliness using the tool DruLiTo. Filters such as Lipinski’s rule were selected to assess the log*P* value, molecular weight, and H-bond acceptors and donors.^23^ The drug likeliness was assessed by installing the tool DruLiTo and uploading the ligand file to the applied filters to yield the scores.

### Swiss ADME Analysis of the Ligand

Every potential drug molecule is required to meet certain criteria in the body such as absorption, distribution, metabolism, and excretion (ADME) to be considered a drug candidate.^24^ Swiss ADME, a computer tool that possesses the models for drug likeliness, physiochemical properties, pharmacokinetics, as well as medicinal chemistry, yields a user friendly output that can be interpreted easily. Apigenin was subjected to Swiss ADME by uploading the SMILES formula to the online tool for evaluation of its suitability as a drug candidate under parameters such as solubility, gastrointestinal (GI) absorption, and bioavailability.^24^

### Preparation of Receptors (Antiapoptotic Proteins)

Cellular activities, such as development, proliferation, differentiation, and specifically elimination of harmful cells, are regulated by a significant pathway called “apoptosis”.^25^ Apoptosis is defined as programmed cell death since it maintains the tissue homeostasis and maintains the health of the tissue.^25^ The two key apoptotic signaling pathways are “intrinsic” and “extrinsic”, in which various proteins play a key role in regulating the mitochondrial outer membrane permeability such as BCL-2 family consisting of various homologues BCL-2, MCL-1,A1/BFL-1, BCL-xl, and BCL-B/BCL-2L10 also known as antiapoptotic proteins and proapoptotic proteins such as BAX, BID, BIM, BIK, etc. The balance in the levels of the antiapoptotic and proapoptotic proteins is crucial to maintain cell health.^25^

Apoptotic proteins such as BCL-2, Bax, BCL-xl, BCL-w, MCL-1, and BRAG-1 were selected for the study as receptors to the ligand of selection. The .pdb files for the above-mentioned proteins were obtained from RCSB Protein Bank with the ids mentioned (Table 1). The .pdb files were made ready for docking with the ligand by eliminating header, inhibitor molecules (native molecule/native ligand molecule), and water molecules which were bound during the crystallization of the receptor molecules. All protein molecules were saved after cleaning in .pdb format for further docking studies.

**Table 1:** RCSB File Numbers of Selected Receptors

s.no	protein/receptors	RCSB PDB IDs
1.	Bcl-2	6QGG
2.	Bcl-w	1ZY3
3.	Bcl-xl	7LH7
4.	Bfl-1	3I1H
5.	Brag-1	6FAE
6.	Mcl-1	5FC4

### Molecular Docking of Apigenin with Antiapoptotic Proteins using CB Dock2

CADD (computer-aided drug discovery) is present day’s crucial tool in identifying the protein–ligand interactions, such as in protein–ligand docking studies.^26,27^ At present, a variety of binding site detecting tools are available which assist in the identification of amino acid residues that are involved in binding with the ligands. Most of these pose a cumbersome process of manually grouping the residues and defining the parameters after multiple docking cycles to obtain the result. This time-consuming process has been addressed by various latest tools that dock blindly and detect the cavity of binding. CBDock2 is one of them that was used in the present study for ligand–protein docking studies.^27,28^

CBDock2 is designed for enhanced blind docking by identifying the binding cavities of the protein in the study and producing the calculated sizes and centers using latest Autodock Vina. For user ease, CBDock2 is enabled with 3D visualization and alignment of Vina scores from lowest to highest. CBDock2 achieved ∼70% similarity of the high-ranking positions and was within 2 Å of the RMSD (root-mean-square deviation) from X-ray crystallography. CBDock2 is freely available at http://cao.labshare.cn/cb-dock/.^4−6^^,^^26−28^

CBDock2 was used to dock the ligand (apigenin) with all antiapoptotic proteins (receptors) BCL-2, BCL-w, BCL-xl, BRAG-1, BFL-1, and MCL-1 using autoblind docking, and the resultant output, i.e., docked .pdb file of protein–ligand complexes, was downloaded based on the lowest Vina score from the various cavities listed. The Vina score, the cavity center coordinates, and the amino acids involved in the bonding between the receptor and the ligand were all recorded as results. The .pdb files were uploaded to Chimera viewing software, and images were captured.

### Identifying the Amino Acids in the Binding Cavity and the Bonds Present between Antiapoptotic Proteins and Ligand using PLIP

PLIP allowed us to easily identify various interactions between biological macromolecules and their ligands to provide atom-level information on the binding characteristics as well as publication-ready visualizations and parable output files. In this study, the .pdb files of the receptors, as obtained from RCSB, were uploaded to the PLIP tool which reported the amino acids present in the interaction of the receptor with its respective native inhibitor along with the nature of interaction; this information was retained (Table 3). A similar process was followed to identify the amino acids present between the receptor and apigenin as well; by uploading the docked .pdb file (downloaded from CBDock2) to PLIP, the results were recorded. The comparison of amino acids present in the binding pocket of the native ligand and apigenin gives us an idea of the similarity in the binding pockets of the native ligand and apigenin even after autoblind docking. This approach is an add-on for the comparative check of the used tool and authenticity of the current docking study.

### Similarity Check of the Amino Acid Residues Present between the Ligand and Native Inhibitor with the Antiapoptotic Proteins

The interacting amino acids and their positions (native receptors) recorded from PLIP as well as the interacting amino acids and their positions of the protein–ligand complex reported from PLIP were compared to identify the similarity. This information is crucial to identify the binding cavity as well as to predict the efficacy of the ligand to occupy the same binding cavity as the inhibitor, which therefore may possess a similar inhibitory effect on the antiapoptotic proteins.

### Energy Minimization

All receptor–ligand complexes were minimized for energy using the software SPDV version 4.10 (Swiss PDV). This tool helps identify the ideal the conformation possessing the minimized energy as well as the accuracy of the molecular docking.^29^ The force field energy values as well as the energy minimized values of apigenin with apoptotic proteins were recorded for comparison in minimization.

### Molecular Simulation Study

Protein–ligand docking essentially docks a ligand into a rigid big molecule (protein) to obtain the prime binding conformation along with the maximum affinity within the protein docking pocket. The protein binding pocket identified by the docking tools is considered most optimal only if it can mimic the native binding conformation of the ligand compared to the crystallized protein with their respective inhibitor molecules; therefore, a standard metric value used to compare the distance between the native pose and the predicted pose is RMSD. To conduct the molecular simulation study, MyPresto v 5.0 standalone software was used.^30^ Briefly, the parameters were set as follows: (a) The global minimization step was set to a loop limit of 5000 with the generalized Born method. (b) The global dynamics was set to perform with 5,000,000 loop limits with (c) 300° K initial and (d) constant temperature and 10 ns time. Rest of all parameters were kept as default. The RMSD as well as the RMSF values of apigenin with all given proteins have been tabulated.

### Cell Line and Cell Culture

The cervical cell line HeLa was purchased from Addexbio (Catno: C0008001). It was cultured using DMEM (Gibco, USA) with 10% FBS (fetal bovine serum) and penicillin-streptomycin (100 μg/mL) and incubated at 5% CO_2_ at 37 °C.

### Preparation of Apigenin Concentrations

Apigenin was procured from MedChem express (cat. no. HY-N1201, CAS No. 520–36–5) and was dissolved in DMSO (Sigma-Aldrich, USA) at 1 mg/50 μL as stock, which was stored at −20 °C. Further dilutions of 5, 10, and 15 μM were prepared in complete DMEM (with 10% FBS) for the treatments.

### Colony Formation Assay

Approximately, 25 × 10^4^ HeLa cells were plated into each well of a six well plate and allowed to attach for 24 h. These cells were treated for 48 h with apigenin (5, 10, and 15 μM) keeping the control wells untreated. The treated cells as well as the control cells were then trypsinized with trypsin (0.25%), and approximately 500 cells were taken from each concentration as well as the control and plated into fresh plates and left for attachment and growth for a fortnight with frequent replenishment of reconstituted media. The colonies formed were then fixed using absolute methanol and stained using crystal violet dye 0.5%), and the images of the wells were captured using an inverted microscope. The colony formation efficiency was calculated using the formula
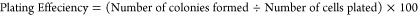


### Microscopic Examination of HeLa Cells Undergoing Apoptosis

HeLa cells were plated at 25 × 10^4^ cells/well in a 12-well plate and treated for 48 h with apigenin (5, 10, and 15 μM) keeping the controls untreated. The images were captured at 40× magnification (Olympus CKX 41, Japan) of cells undergoing apoptosis in the treated wells and compared with untreated cells.

## Results

### Apigenin Exhibits Druglike Properties

Drug likeliness evaluation of apigenin was recorded using the bioinformatics tool DruLiTo as well as Swiss ADME, both of which have yielded results in accordance with the requirements for a molecule to be considered a drug as suggested by Lipinski’s rule of 5, in which the molecule should not possess more than 5 hydrogen bond donors, no more than 10 hydrogen bond acceptors, molecular weight not more than 500 Da, and finally partition coefficient not greater than 5, all of which are reported to be fulfilled by apigenin (Table 2). According to Swiss ADME, the molecule’s solubility in water is recorded as the log*S* value which is interpreted as ≤10 poorly soluble, ≤6 moderately soluble, < =4 soluble, ≤2 very soluble, and < =0 highly soluble. Apigenin reports a log*S* value of −4.40 falling into the highly soluble category. Apigenin also reports high GI absorption, which is a crucial aspect for a molecule to qualify as a drug. The bioavailability score for apigenin was found at 0.55 (Table 2).

**Table 2:** Drug Likeness Parameters

s.no	parameter	result
1.	Log*P*^a^	1.13
2.	HBA [Hydrogen bond acceptor]^a^	5
3.	HBD [Hydrogen bond donor]^a^	3
4.	Molecular Weight^a^	270.05
5	Log*S* (All)^b^	–4.40
6	Solubility in H_2_O^b^	Moderately Soluble
7.	G.I Absorption^b^	High
8.	Bioavailability Score^b^	0.55

a Calculated by DruLiTo.

b Calculated by Swiss ADME.

### Molecular Docking of Apigenin with Antiapoptotic Proteins

**Figure 2:**
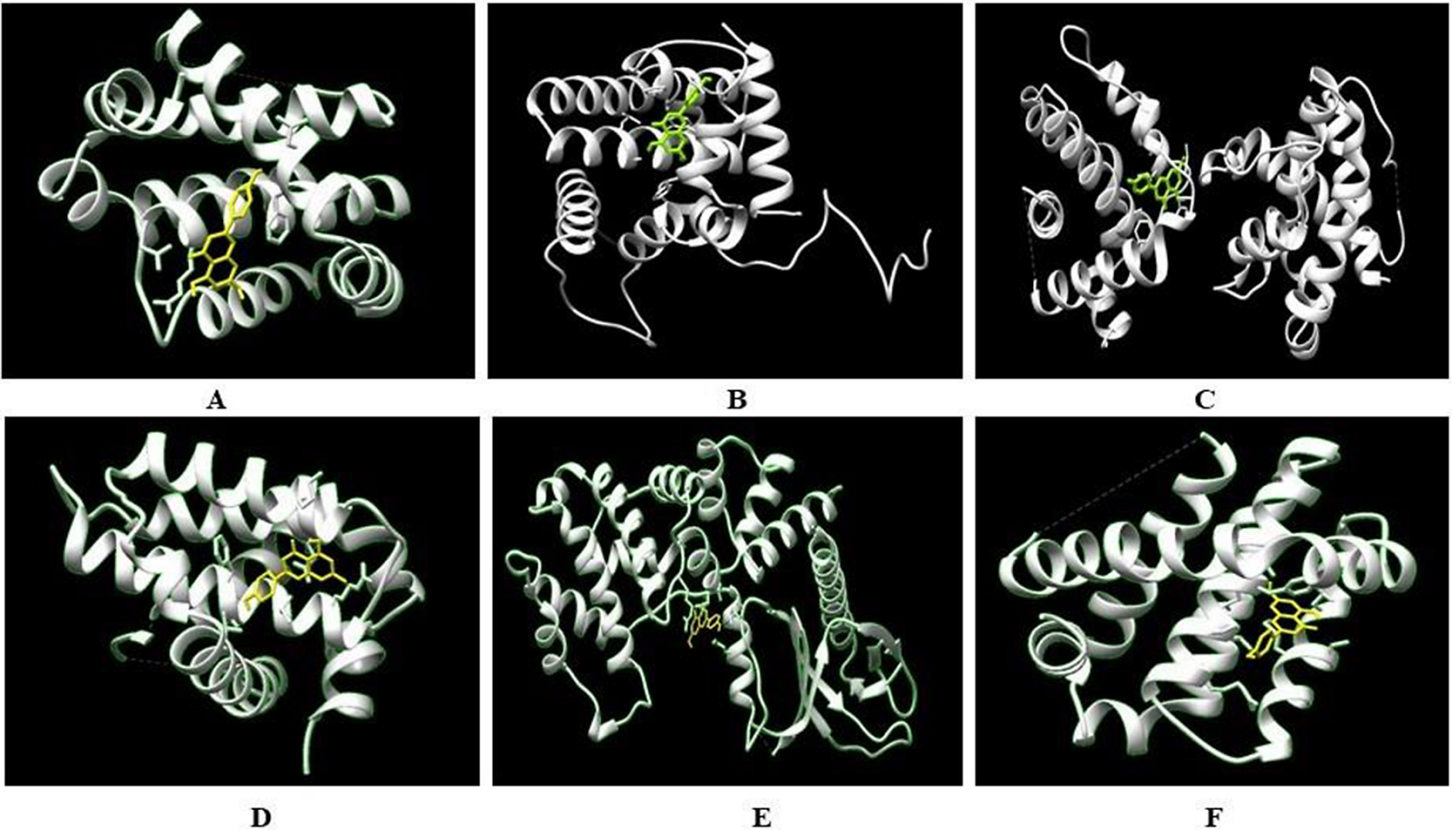
(A) Bcl-2 protein binds with ligand apigenin, (B) Bcl-w protein binds with ligand apigenin, (C) Bcl-xl protein binds with ligand apigenin, (D) Bfl-1 protein binds with ligand apigenin, (E) Brag-1 protein binds with ligand apigenin, and (F) Mcl-1 protein binds with ligand apigenin.

Docking studies helped to predict the interaction of antiapoptotic proteins with Apigenin. Docking also yielded the orientation or cavity where apigenin bound to the receptors with the lowest energy and highest binding affinity. Apigenin was docked into the apoptotic proteins (BCL-2, BCL-xl, BCL-w, BFL-1, MCL-1, and BRAG-1) using CBDock2 (Figure 2). The binding cavity with the lowest energy (Vina score) was selected, and the coordinates of each of the interactions were recorded along with the specific amino acids binding with the ligand (Table 3). BCL-2 and BCL-xl have shown multiple similarities in the amino acid residues binding with apigenin and their native inhibitor, such as apigenin in complex with BCL-2 was found to interact with residues Asn143, Arg146, Phe104, Tyr108, as well as Val148 which also bound similarly with their native ligand in the original structure retrieved form RCSB.pdb file. Similarly, BCL-xl is reported to bind Ser106, Leu108, Phe97, Ala104, Phe97, and Leu108 amino acid residues with the native ligand as well as apigenin. BCL-w possessed no native heteroatom in the crystalline structure obtained from RCSB, and therefore comparison was not possible. A hydrogen bond at Leu67 along with various interactions at Leu63, Ala64, Phe78, and Phe101 were recorded in the complex of apigenin with BCL-w. The .pdb files of the protein–ligand complexes were saved for further analysis. BFL-1 was found to be interacting with apigenin through amino acid residues Arg88, Val48, Val74, Glu78, and Phe95. BRAGG-1 was interacting with Apigenin at amino acid residues Asp594, Asp595, Ser598, Gln601, Gln450, Val597, and Pro617 as compared to the amino acids of native heteroatom binding with BRAG-1 at Lys73 and Arg565. MCL-1 is reported to bind with apigenin through amino acids Leu246, Val253, Thr266, Leu26, and Phe270 as compared to their binding with their native heteroatom at Phe319. (Figure 2).

**Table 3:** Vina Score and Amino Acid Interaction of Apigenin in Complex with Proteins and Their Native Heteroatoms

proteins/receptors	energy/vina score	docking coordinates (CB Dock)	interacting amino acids with apigenin (PLIP)	hydrogen bonds between apigenin and protein (PLIP)	amino acids involved in the binding of the native protein with its heteroatom (PLIP)
BCL-2(6QGG)	–7.2	–15, 17, −6	Phe100, Asn143, Arg146, Phe104, Tyr108, Arg46 Val148	Phe100, Asn143, Arg146	Asn143, Trp144, Gly145, Tyr202, Phe104, Tyr108, Met115, Val133, Leu137, Arg146, Val148, Phe153, Tyr202, Asp103, Asp111.
Bcl-w (1ZY3)	–7.8	–8, −9, −4	Leu67, Leu63, Ala64, Phe78, Phe101	Leu67	No native ligand was found on the crystalline structure on RCSB
Bcl-xl (7LH7)	–7.2	7, −11, −11	Arg103, Ser106, Phe97, Arg102, Ala104, Leu108, Phe146	Arg103, Ser106	Ser106, Leu108, Asn136, Gly138, Arg139, Phe97, Tyr101, Ala104, Phe105, Leu108, Thr109, Leu130, Val141, Phe146, Ala149, Tyr195
Bfl-1 (3I1H)	–6.7	4, −10, −2	Arg88, Val48, Val74, Glu78, Phe95	Arg88	Hetero atom is polypeptide helix hence, PLIP could not find the type of interactions.
Brag-1 (6FAE)	–7.6	2, 71, −33	Asp594, Asp595, Ser598, Gln601, Gln450, Val597, Pro617	Asp594, Asp595, Ser598, Gln601.	The native binding site is different than the one predicted by PLIP. PLIP reports multiple hydrogen bonds with Apigenin, which help us to predict the ligand- receptor interaction is firm, which is supported by low Vina score as well as molecular simulation study.
Mcl-1 (5FC4)	–7.5	4, 4, 14	Leu246, Val253, Thr266, Leu267, Phe270.	Nil	Phe 319

**Figure 3:**
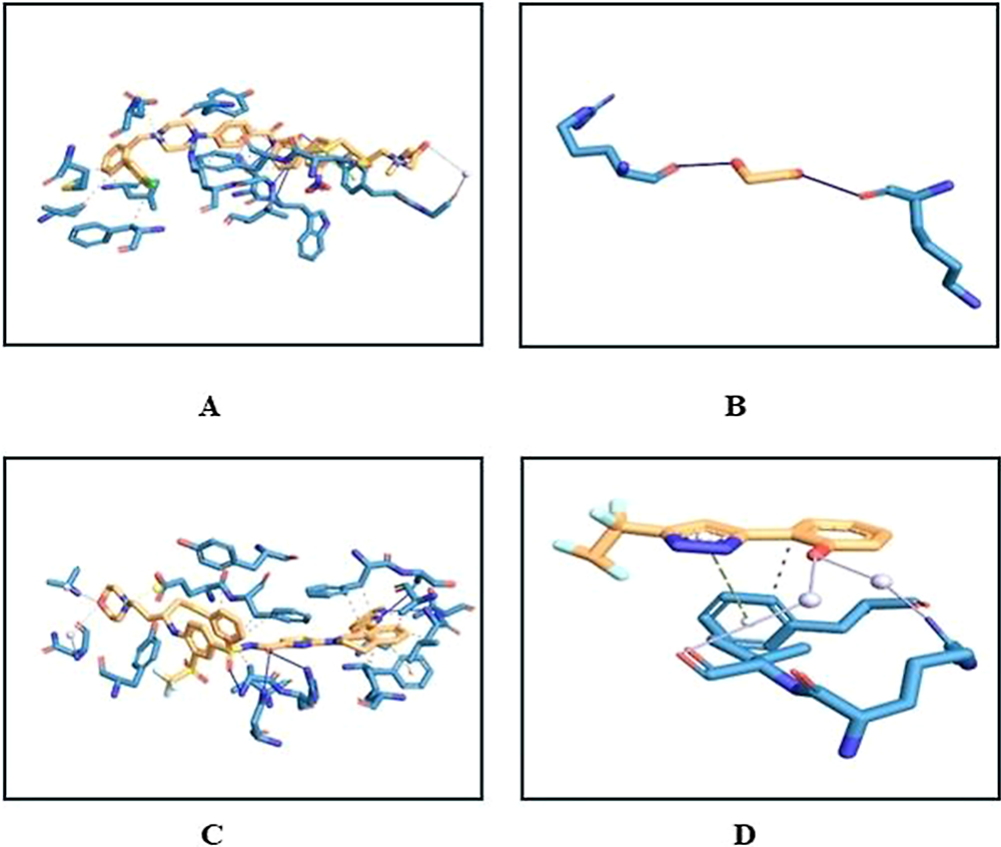
Amino acid side chain interaction with the native ligand of apoptotic proteins in the native binding site by PLIP tool (A, Bcl-2; B, Brag-1; C, Bcl-xl; and D, Mcl-1).

### Chemical Bonds Identified in the Interaction of Apoptotic Proteins and Apigenin

**Figure 4:**
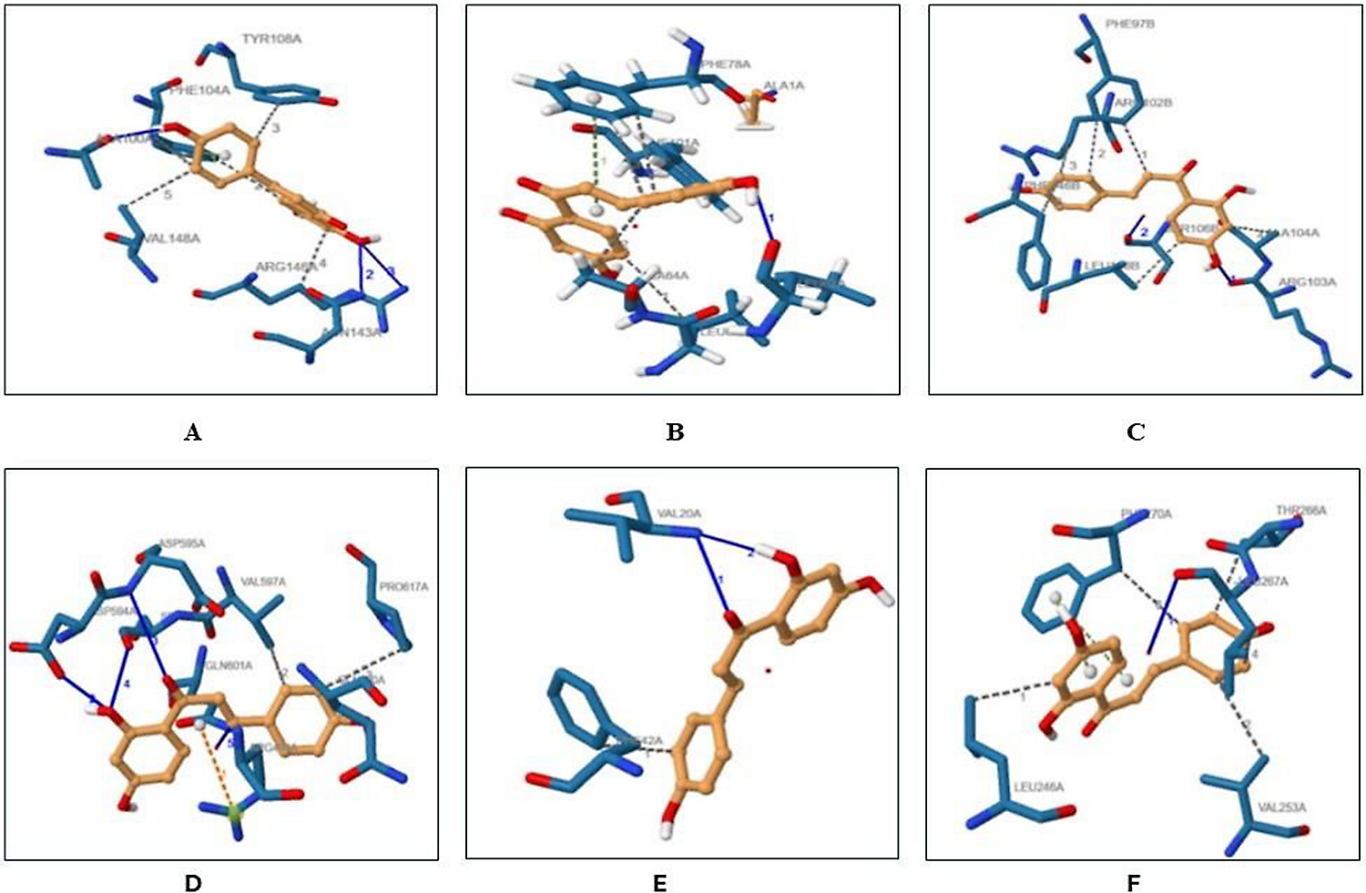
Bonds present between apoptotic proteins and the ligand (apigenin) by PLIP tool: (A) Bcl-2, (B) Bcl-w, (C) Bcl-xl, (D) Bfl-1, (E) Brag, and (F) Mcl-1.

PLIP is an online tool that helps identify the bonds present between the ligand (apigenin) and receptors (apoptotic proteins), as well as the strength and nature of the bonds. PLIP was used to record the nature of various bonds present between apigenin and apoptotic proteins (Figure 4) (Table 3) as well as the bonds present between the receptors and their respective native inhibitory molecules (Figure 3). A number of hydrogen and hydrophobic interactions were recorded. BCL-2 reported three hydrogen bonds at Phe100, Asn143, and Arg146 as well as four hydrophobic interactions Phe104, Tyr108, Arg146, and Val148 with apigenin. BCL-w possessed one hydrogen at Leu67 and hydrophobic bonds at Leu63, Ala64, Phe78, and Phe101 with apigenin. BCL-xl made two hydrogen bonds at Arg103 and Ser106 and five hydrophobic interactions at Phe97, Arg102, Ala104, Leu108, and Phe146 in complex with apigenin.

BFL-1 was found to interact with apigenin with one hydrogen bond at Arg88 and four hydrophobic interactions at Val48, Val74, Glu78, and Phe95. BRAG-1 possessed four hydrogen bonds at Asp594, Asp595, Ser598, and Gln601 and three hydrophobic interactions at Gln450, Val597, and Pro617. MCL-1 made five hydrophobic bonds at Leu246, Val253, Thr266, Leu267, and Phe270 with apigenin. The amino acids present in these binding sites along with their position were noted (Table 3) for comparison.

### Energy Minimization of Apoptotic Proteins–Apigenin Complexes

Energy minimization of docked (Apigenin and apoptotic protein complexes) files was performed using the SwissPDB Viewer (SPDBV). The energy minimization of the docked molecules was performed to yield the set of coordinates that possess minimum energy conformation of the protein–ligand complex (Table 4). Upon energy minimization, all of the complexes have shown a remarkable decrease in their energies. BRAG-1 reported the maximum decrease in the energy post minimization of −Δ5754.18 kJ/mol followed by BCL-xl which reports a decrease of −Δ3377.7 kJ/mol, BCL-2 −Δ2655.8 kJ/mol, MCL-1 −Δ2614.3 kJ/mol, BCL-w −Δ2152.4, and finally BFL-1 −Δ2023.9 kJ/mol (Table 4).

**Table 4:** Force Field Energies Compared to the Minimized Energies

ligand	protein	energy force field (kJ/mol)	energy minimized (kJ/mol)
	BCL2	–5596.129	–8251.93
	BCL-W	–6627.071	–8779.45
Apigenin	BCL-XL	–13,417.518	–16,795.25
	BFL-1	–6278.688	–8315.51
	BRAG-1	–17,524.65	–23,278.781
	MCL-1	–5985.726	–8600.972

### Molecular Simulation of Antiapoptotic Proteins–Apigenin Complexes

The RMSD and RMSF values of the docked complexes were obtained using MyPresto v 5.0 for molecular simulation. The simulation is used to assess the stability of the protein–ligand complexes under physiological conditions. The molecular simulation was performed for 10 ns for all molecules to yield the RMSD and RMSF values (Table 5). BCL-2 in complex with apigenin reported an RMSF value of 3.075728 × 10^01^ and an RMSD value of 3.449541 × 10^00^, while BCL-w reported an RMSF value of 3.106904 × 10^01^ and an RMSD value of 7.892349 × 10^00^. BCL-xl reported an RMSF value of 2.994649 × 10^01^ and an RMSD value of 3.219422 × 10^00^, and BRAG-1 as well reported an RMSF value of 3.003354 × 10^08^ and an RMSD value of 7.552535 × 10^00^. BFL-1 reported an RMSF value of 3.009402 × 10^01^ and an RMSD value of 2.543095 × 10^00^, and MCL-1 reported an RMSF value of 2.917332 × 10^01^ and an RMSD value of 4.777887 × 10^00^.

**Table 5:** RMSD and RMSF Values for Apigenin with Apoptotic Proteins

ligand	protein	RMSF value	RMSD value
	BCL-2	3.075728E+01	3.449541E+00
	BCL-W	3.106904E+01	7.892349E+00
Apigenin	BCL-XL	2.994649E+01	3.219422E+00
	BRAG-1	3.003354E+08	7.552535E+00
	BFL-1	3.009402E+01	2.543095E+00
	MCL-1	2.917332E+01	4.777887E+00

**Figure 5:**
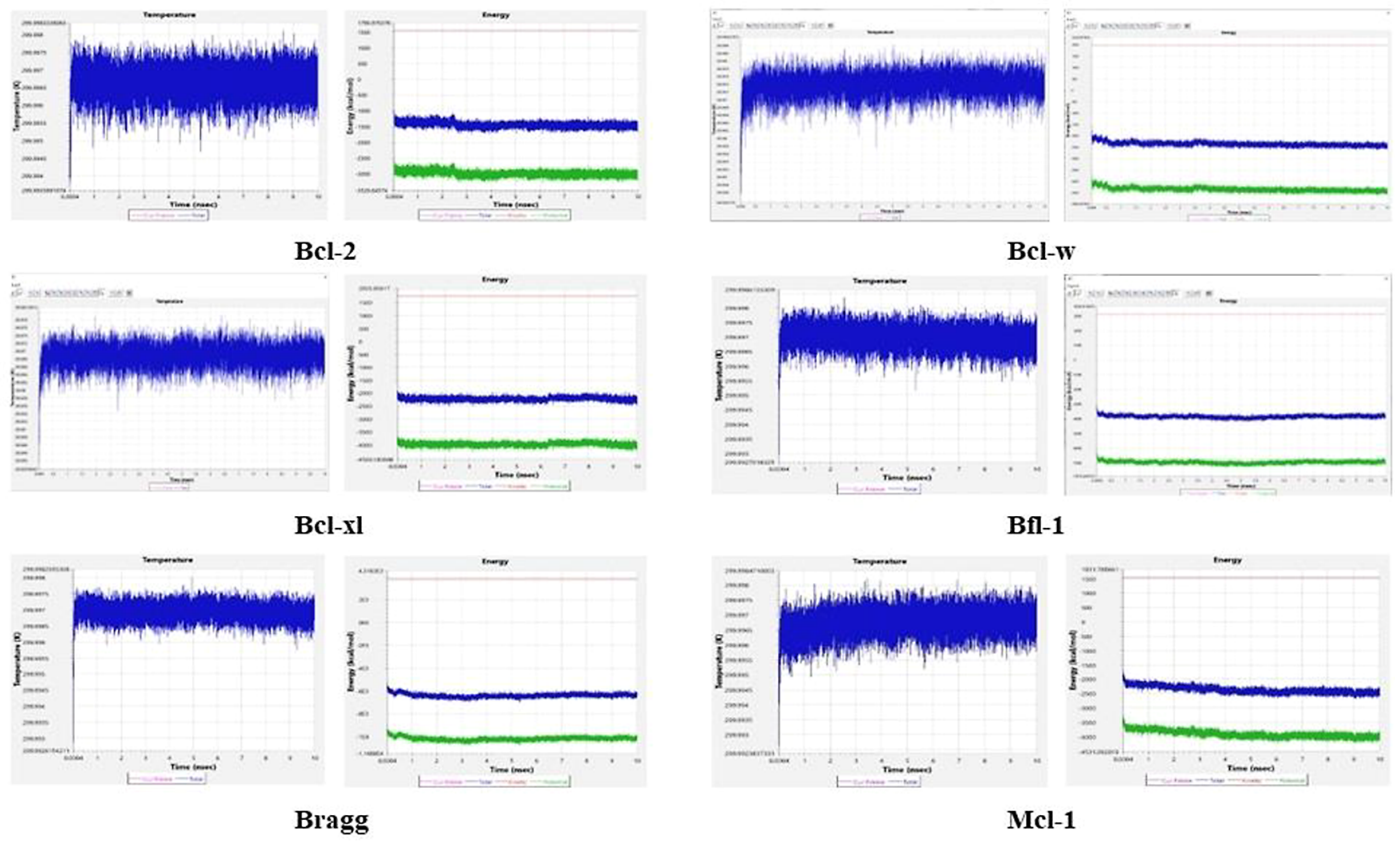
Energy and temperature graphs of antiapoptotic proteins–apigenin complexes.

RMSD and RMSF values help us to understand how the molecules interact with the ligand to attain the most stable conformation and equilibrium through the passage of time. Distorted energy or temperature graphs of a complex under molecular simulation imply that the protein–ligand complex is unstable under simulated physiological conditions. The energy and temperature graphs for each protein with apigenin during simulation reported stability throughout the 10 ns, confirming the equilibrium during the simulation and proving the stability of intermolecular interaction between the antiapoptotic proteins and the ligand (Figure 5).

### Apigenin Curbs Colony-Forming Capability in HeLa Cells

**Figure 6:**
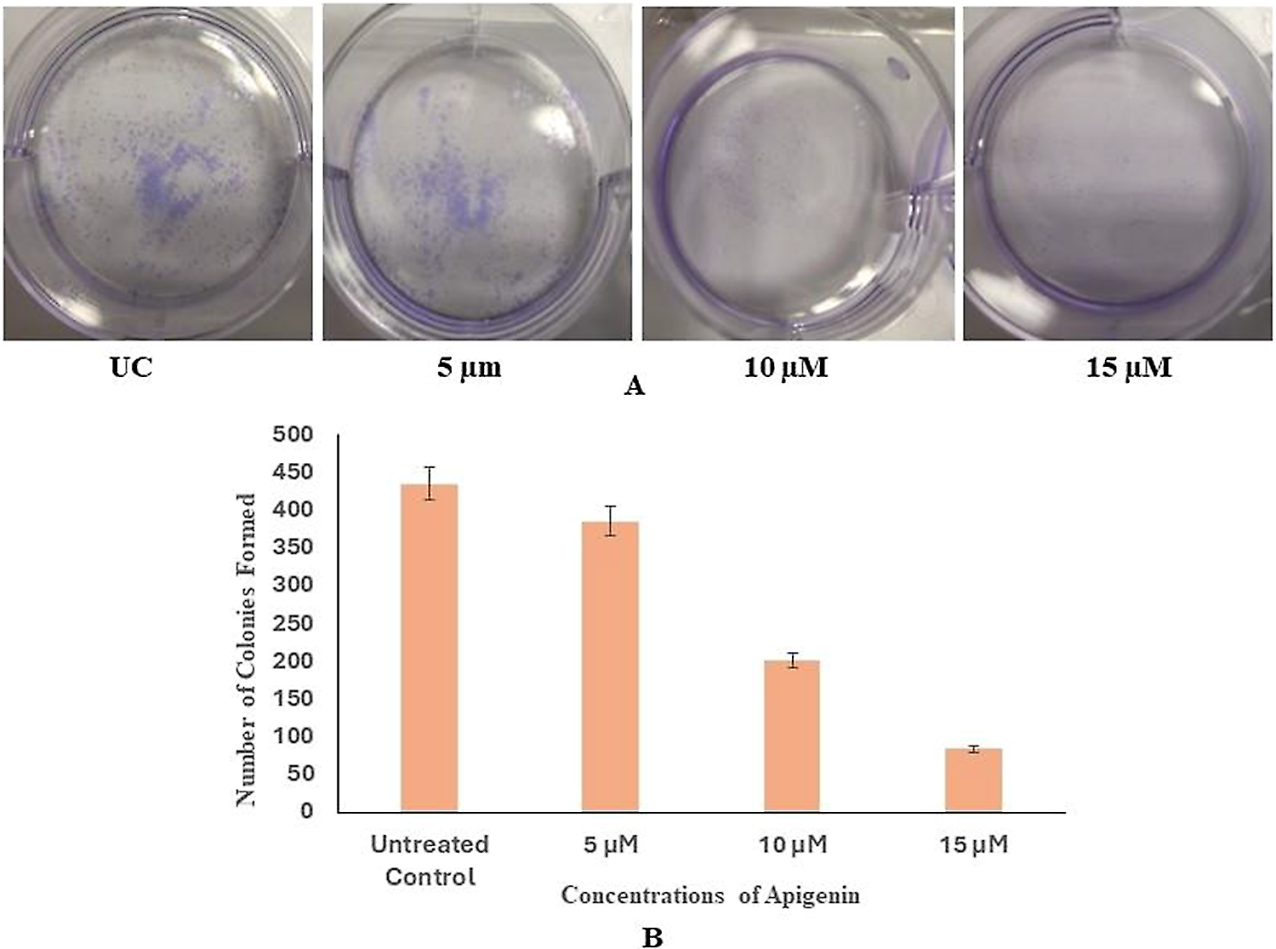
Colonies formed after treatment with apigenin. (A) Untreated control, 5, 10, and 15 μM. (B) No. of colonies plotted in the graph.

Colony formation efficiency of treated HeLa cells was performed and yielded the following result in which the colony-forming efficiency of the HeLa cells was seen to reduce with increasing concentrations of apigenin (5, 10, and 15 μM) from 520 colonies in control wells to 75 colonies at the highest concentration, i.e., 15 μM (Figure 6A). The formula below was used to calculate the plating efficiency, and the graph was plotted using the values obtained (Figure 6B). This suggests that apigenin has a profound effect on the proliferative capacity of the HeLa cells.
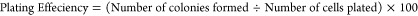


### Apigenin Induces Apoptotic Characteristics in HeLa Cells

**Figure 7:**
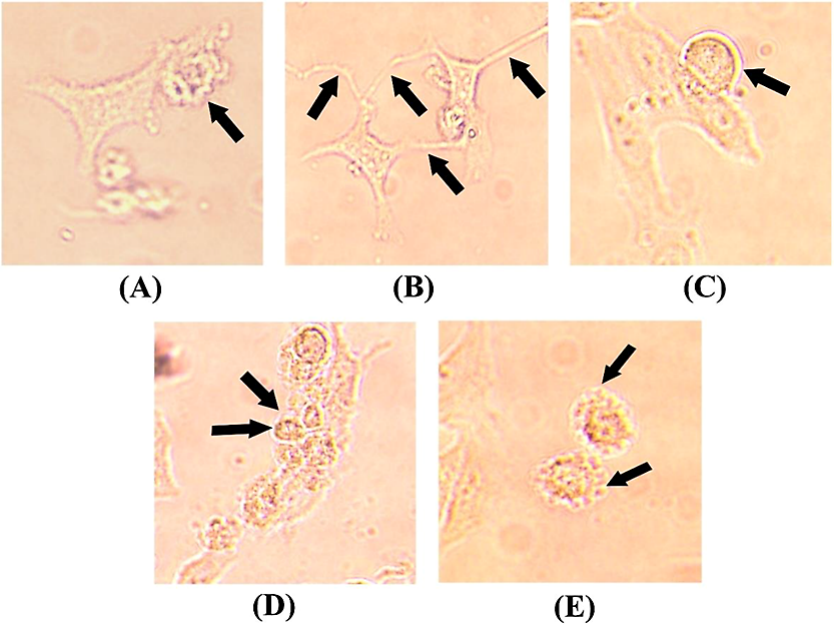
Characteristic traits of apoptosis following the treatment of HeLa cells with apigenin (40× magnification). (A) Blebbing, (B) spike formation, (C) cytoplasmic oozing, (D) nuclear fragmentation, and (E) surface blisters.

Following the treatment of HeLa cells with apigenin, the cells were observed under the microscope for characteristic traits of apoptosis such as spike formation in cells, nuclear fragmentation, oozing of cytoplasmic content, and blebbing. All of these traits were observed in the treated cells, indicating that apigenin induces apoptosis (Figure 7).

## Discussion

Apoptosis, the principal mechanism responsible for maintaining tissue homeostasis by eliminating the damaged cells and mediating the cell (via pro- and antiapoptotic proteins), is a crucial hallmark of cancer since the proteins involved are deregulated in cancers leading to the division of cells with damaged or mutated DNA. Evading apoptosis is one of the vital signals of a cancerous cells. Treatments available for cancer are known to be nontargeted, producing severe cytotoxicity and leading to adverse effects to the human body; therefore, the need for a least cytotoxic, natural, and selective treatment led to identify the bioactive compounds present naturally in vegetables and fruits. These compounds are classified into various groups based on the chemical structure. Apigenin, a compound belonging to the flavonoid group, has been identified for this study, Apigenin has reported various anticancer properties such as reinstating apoptosis, anti-inflammatory, and antioxidation.^15−18,31^ Hence, apigenin was studied for its druglike properties using bioinformatics tools, such as DruLiTo and Swiss ADME.

DruLiTo and Swiss ADME are tools used to examine any small molecule for its suitability as a potential drug. Current study showed that apigenin is radially soluble in water with log*S* as −4.40 and good at absorption in GI (0.55), fulfilling the criteria of Lipinski rules, theoretically. Earlier studies of refs (31,32) have reported similar findings while using Swiss ADME for apigenin’s suitability as a drug. Findings of the present study agree with the other studies of apigenin which have shown its potential as a drug candidate for further studies.^24,33^

RCSB protein data bank is a global archive for 3D structures of biomolecules, especially proteins. RCSB also provides the structural coordinates of proteins in .pdb format which refer to their tertiary and quaternary structures. Those structures for the proteins assessed in this study were downloaded. For performing the docking studies of small molecules with proteins, structural coordinates of both the molecules are required in .pdb format.^26,27^ To yield the .pdb files of small molecule (apigenin), its respective SDF file^26,27^ was obtained from PubChem repository. The SDF file was converted into .pdb file format (3D canonical) by using Open Babel tool.^34^ The native binding site on the proteins was worked out (involved amino acids) for all studied proteins except BFL-1 as well as BCL-w using the PLIP tool,^35^ while the best suitable binding conformation after binding apigenin with the proteins with the highest binding energy (Vina score) was calculated using CBDock2. CBdock2 finds the best suitable binding site through blind docking.^27^ Hence, it was reported that the binding site predicted by CBDock2 has the lowest Vina score.

After docking analysis, it was found that BCL-2 showed multiple similarities in the amino acid residues found interacting with its native heteroatom and apigenin (Asn143, Arg146, Phe104, Tyr108, as well as Val148) followed by BCL-xl. The native binding site and the binding site for apigenin shares multiple similar amino acid residues (Ser106, Leu108, Phe97, Ala104, Phe97, and Leu108), suggesting that the inhibitory capability of apigenin is similar to its native heteroatom. In the case of BCL-w, no native ligand was found bound to the protein in the structure downloaded from RCSB, and therefore a native binding cavity was not available; however, the binding of BCL-w and apigenin was considered for reporting due to the presence of 1 hydrogen bond at amino acid Leu67 along with the molecular simulation results that show considerable stability in the energy and temperature over the time period of 10 ns simulation (Figure 8) (Table 3). Similarly, the crystal structure of BFL-1 does not show any binding site for small molecules, although that had a binding site for the polypeptide (heteroatom). Hence, we did not consider the polypeptide binding site as a binding cavity suitable for small molecules like apigenin. However, docking reported a hydrogen bond at amino acid residue Arg88 along with various other interactions at Val48, Val74, Glu78, and Phe95 between apigenin and BFL-1.^36^ Similarly, BRAG-1 reports no similar amino acid residues in the binding site between its native heteroatom and apigenin, although multiple hydrogen bonds such as Asp594, Asp595, Ser598, and Gln601 were reported to be present between apigenin and BRAG-1. In this case, four hydrogen bonds with apigenin as compared to two hydrogen bonds with their native heteroatom suggest a stronger binding between apigenin and BRAGG-1 as compared to the native heteroatom, indicating the stronger inhibiotory capability of apigenin compared to its native heteroatom. MCL-1 does not report any similar amino acid residues in the binding site of apigenin or its native heteroatom. Amino acid residues Leu246, Val253, Thr266, Leu267, and Phe270 were found involved in binding with apigenin.

Minimum energy is the most stable state of any system. Here, the protein and the small-molecule complex structure are the concerned system. Energy minimization is a key step to finding a configuration which is stable and minimized locally, and an energy minimized structure will provide a clear idea of the orientation of the active site residues, size of the active site cavity, etc.^23^ In the present study, all energy minimizations of protein-small molecule was performed using SPDBV.^37^ Post minimization, the results reported a noticeable decrease in the energy of the concerned molecular complexes, which suggests that the apoptotic protein-apigenin complexes are stabilized well.

Molecular dynamics is a tool that assesses the molecular systems for their dynamics at the atomic level.^38^ The RMSD and RMSF values are considered the index of stability of complex structures. The RMSD values of BCL-w 0.7892349E, BCL-xl 0.3219422, BCL-2 0.3449541, BFL-1 0.316, MCL-1 0.478, and BRAG-1 0.755 suggest that the complex remained stable over the passage of 10 ns (Figure 8). Due to several calculations and the requirement of higher configuration in computers, which are crucial for molecular simulation studies, in this study, we have considered a 10 ns frame of time.

The current study provided considerable *in silico* evidence for apigenin to be considered as a suitable candidate for further studies as a drug (high GI absorption, high solubility (log*S*), bioavailability, and nonviolation of any of the Lipinski rules). In addition, apigenin was found to bind in the same/similar binding pocket in which the native ligands of protein bind, especially for BCL-XL, BCL-w, and Bcl-2. Also, the apigenin-protein complexes had shown stability during the molecular simulation studies. Collectively, the present study predicts that (a) apigenin is a suitable candidate as a drug and (b) could exert a similar inhibitory effect as the native ligands of studied proteins. Hence, by considering these computational predictions in the current study, apigenin was used to test in vitro.

The in vitro studies for assessment of apigenin’s ability as a drug to induce antiproliferation through apoptotic reinstatement were performed through clonogenic assay as well as microscopic examination for characteristic apoptotic features. The clonogenic assay revealed a significant decrease in the number of colonies formed post treatment with apigenin. Hence, this finding suggests that apigenin has a remarkable effect on the proliferative capability of HeLa cells. A microscopic examination of the treated cells has revealed the different changes in the morphology of the HeLa cells.^39^ Such an apoptotic morphology includes nuclear blebbing, nuclear fragmentation, oozing of cytoplasmic content, spike formation, etc. (Figure 7). Microscopic morphological evidence indicates that apigenin can reduce the proliferative capability in HeLa cells by inducing apoptosis. From the present study and evidence, a deeper insight into apigenin’s capability as a drug in modulating proliferation, apoptosis, and migration of cancer cells has been obtained. However, to establish this property and consolidate apigenin’s candidature as a cancer drug, furthermore *in silico* and in vitro studies are warranted.

## Conclusions

This computational study provides an insight into apigenin’s ability to interact with antiapoptotic proteins Bcl-2, Brag-1, Bcl-xl, Mcl-1,etc. This study showed that apigenin interacts with the concerned protein with higher affinity and higher stability (lowest energy) as compared with their native small molecules. This suggests higher inhibitory capability of apigenin on the antiapoptotic proteins which are known to be upregulated in cancers causing the cells to evade apoptosis. Since these protein native molecules had been studied and considered antitumor, apigenin, while interacting more radially in this computational study, could be also regarded as antitumor. This consideration was tested with in vitro study through clonogenic assay which reported the decrease in the proliferative capability of HeLa cells possibly due to the inhibition of antiapoptotic proteins by apigenin as observed in the *in silico* study. The observed morphological changes in HeLa cells that are characteristic features of the apoptotic cells suggested that apigenin is capable of inducing apoptosis in a dose-dependent manner and may be considered as a potential antitumor drug candidate.
